# Background and Rationale — CDC Guidance for Communities Assessing, Investigating, and Responding to Suicide Clusters, United States, 2024

**DOI:** 10.15585/mmwr.su7302a1

**Published:** 2024-02-29

**Authors:** Michael F. Ballesteros, Asha Z. Ivey-Stephenson, Eva Trinh, Deborah M. Stone

**Affiliations:** 1 Division of Injury Prevention, National Center for Injury Prevention and Control, CDC

## Abstract

*To assist community leaders in public health, mental health, education, and other fields with developing a community response plan for suicide clusters or for situations that might develop into suicide clusters, in 1988, CDC published *Recommendations for a Community Plan for the Prevention and Containment of Suicide Clusters (MMWR Suppl 1988;37[No. Suppl 6]:1–12).* Since that time, the reporting and investigation of suicide cluster events has increased, and more is known about cluster risk factors, assessment, and identification. This supplement updates and expands CDC guidance for assessing, investigating, and responding to suicide clusters based on current science and public health practice. This report is the first of three in the MMWR supplement that describes an overview of suicide clusters, information about the other reports in this supplement, methods used to develop the supplement guidance, and the intended use of the supplement reports. The second report, *CDC Guidance for Community Assessment and Investigation of Suspected Suicide Clusters — United States 2024*, describes the potential methods, data sources and analysis that communities can use to identify and confirm suspected suicide clusters, and better understand the relevant issues. The final report, *CDC Guidance for Community Response to Suicide Clusters — United States, 2024*, describes how local public health and community leaders can develop a response plan for suicide clusters. The guidance in this supplement is intended as a conceptual framework that can be used by public health practitioners and state and local health departments to develop response plans for assessing and investigating suspected clusters that are tailored to the needs, resources, and cultural characteristics of their communities.*

## Introduction

In 2021, approximately 48,000 lives were lost to suicide in the United States (*1*). During this time, suicide was among the 10 leading causes of death among persons aged 10–64 years and the second leading cause of death among children and adolescents aged 10–14 and adults aged 25–34 years. Suicide rates peaked in 2018, followed by two consecutive years of declines (5%) during COVID-19; during 2020–2021, rates nearly rebounded to the 2018 peak (*1*,*2*). Age-adjusted rates increased approximately 36% from 10.4 suicides per 100,000 population in 2000 to 14.1 in 2021 (*1*,*3*). Many more persons think about or attempt suicide. In 2021, a total of 12.3 million U.S. adults reported serious thoughts of suicide, 1.7 million attempted suicide (*4*), 22% of high school students seriously considered suicide, and 10% attempted suicide (*5*).

When a group of suicides or suicide attempts occur closer together in time, space, or both than would normally be expected in a community, they are defined as a suicide cluster (*6*,*7*). Suicide clusters are rare and are believed to comprise only a small proportion of overall deaths by suicide; for example, in the United States, an estimated 1%–2% of teenage suicides are part of clusters (*8*). However, suicide clusters can have unique characteristics and challenges and, when they occur, are often highly publicized and can have considerable negative effects on the community, including prolonged grief and elevated fear and anxiety about further deaths (*9*,*10*).

## Overview and Types of Suicide Clusters

Suicide clusters have been reported in diverse populations and settings including psychiatric inpatients (*11*,*12*), teenagers and young adults (*8*,*13*–*15*), schools (*16*–*18*), prison inmates (*19*,*20*), and American Indian and Native American communities (*21*–*25*). The two most commonly reported types of suicide clusters are point clusters and mass clusters. Point clusters (or spatial-temporal clusters) represent a greater-than-expected number of suicides or suicide attempts that occur within a time period in a specific location (https://www.cdc.gov/suicide/resources/suicide-clusters.html). Point clusters might occur in a community/county or an institution such as a school, university, or psychiatric inpatient setting. Mass clusters (or temporal clusters) represent a greater-than-expected number of suicides or suicide attempts spread out geographically within a time period (https://www.cdc.gov/suicide/resources/suicide-clusters.html).

The causes of suicide clusters are not well understood. Available reports of point clusters tend to only describe the characteristics of decedents involved in the cluster and are not designed to rigorously assess risk. Persons involved in point clusters tend to be male and adolescents or young adults and have a history of substance use, self-harm, and mental illness (*7*,*26*–*28*). Risk factors for point clusters are postulated to be the same as general risk factors for suicide (*28*) and therefore do not aid in identifying those most at risk for becoming part of a suicide cluster.

Certain methodological barriers have been identified that preclude better understanding of cluster risks. These barriers include selection bias in the available reported clusters, limited opportunities for comparison groups, relatively small numbers of suicides in diverse populations, and the absence of a standard definition for time and space parameters (*28*,*29*), which make combining or comparing individual case studies challenging. In addition, no standard analytic approach exists to test whether the observed number of suicides is greater than expected. Several different methods have been used, including the application of spatial statistics using geographic information systems, and statistical methods such as Knox, Poisson, and Scan tests (*30*–*32*). These methods can individually serve the organizations and communities experiencing potential clusters but also complicate fully understanding the overall risk for clusters.

Although understanding and evidence of what triggers suicide clusters is lacking, suicide clusters, especially mass clusters, might occur through a process of contagion (i.e., when the exposure to the suicide or suicidal behavior of one or more persons influences others to attempt suicide) (*26*). An exposure can be direct by having a personal connection to the person who has died by suicide, or indirect through media reporting or social media posts about a person who was not a personal connection (*28*). Media influence can be both a risk and protective factor depending on its duration, prominence of source, messaging, and extent of coverage (*33*,*34*).

Media reporting of suicides might be a risk factor when it unintentionally influences increases in suicides, particularly in reporting that mentions the suicide method in the headline and in the text, and includes a statement that suicide is inevitable (*35*). When similar suicides occur after this type of media reporting, the increase might be attributed to the “Werther effect” (also called copycat behavior) (*34*,*36*,*37*). Media influence can relate to point clusters as well as mass clusters. For example, extensive and prominent news coverage of suicides has been reported to play a role in the emergence of point clusters among youth (*33*). Several reports have documented increases in suicide rates following media reports of high-profile celebrities, who might be considered models for imitation (*38*–*41*). In addition, there might be unintended negative consequences of entertainment media portrayals of suicide that do not adhere to best practices for safe reporting (*42*,*43*).

Conversely, responsible media reporting of suicide can be a protective factor and make a positive contribution to prevention efforts by educating the public about coping strategies and treatment (“Papageno effect”) (*34*,*44*,*45*). Accepted best practices (https://reportingonsuicide.org) for reporting on suicide include reporting suicide as a public health problem, including resources (e.g., hotline information and treatment options), providing warning signs, using appropriate language (e.g., “died by suicide” instead of “committed suicide”), emphasizing help and hope, and including information from suicide prevention or mental health experts, and providing resources, such as the recently updated 988 number for the national Suicide & Crisis Lifeline (https://988lifeline.org).

## About this Supplement

CDC has developed new expanded guidance for investigating and responding to potential suicide clusters by using updated information from the literature on suicide clusters, input from subject matter experts, and experiences of public health practitioners and others involved in a cluster identification. The second report in this supplement, *CDC Guidance for Community Assessment and Investigation of Suspected Suicide Clusters–United States, 2024* (*46*), describes for communities the potential methods and data sources that can be monitored for suicide clusters or be further analyzed to confirm suspected suicide clusters and builds on the 1990 CDC Guidelines for Investigating Clusters of Health Events (*47*), which considered clusters of noninfectious diseases, injuries, birth defects, and previously unrecognized syndromes or illnesses, but frames its content and guidance to suicide clusters, which have unique characteristics and challenges. The third report, *CDC Guidance for Community Response to a Suicide Cluster–United States, 2024* (*48*), describes guidance to assist local public health and community leaders on how to develop a community response plan for suicide clusters. This supplement updates and expands the guidance from the 1988 CDC document (*6*).

## Methods

To gather information for the second and third reports in this supplement, in September 2021, staff members from the Division of Injury Prevention in CDC’s National Center for Injury Prevention and Control including behavioral scientists, epidemiologists, and developmental psychologists, with support from a contracted consultant team, Ross Strategic, initiated activities that included a literature review, environmental scan, media review, and input from subject matter experts in the field.

### Literature Review, Environmental Scan, and Media Review

The CDC team conducted a literature review of suicide cluster research to determine the latest science on suicide cluster identification, risk and protective factors, opportunities for using social media as a tool for prevention and response, and best practices and challenges for identifying and responding to suspected clusters. The team searched the English-language published literature via PubMed, Google Scholar, ProQuest, and JSTOR. The following keywords were used to search English language journals: “suicide clusters” OR “suicide cluster” OR “suicide contagion” and “risk factors” or “identifying” or “protective factors” or “prevention” or “containment” or “demographics” or “social media” or “media.” For cluster identification and social media publications, searches went back in time as far as possible with the earliest paper included published in 1990; however, for risk and protective factors, and prevention and response, the search as restricted to publications after 2000 to focus on the most recent reports. No geographic restrictions were placed on articles included. In addition, the team included articles from the CDC’s Suicide, Suicide Attempt, or Self-Harm Clusters website (https://www.cdc.gov/suicide/resources/suicide-clusters.html) and articles provided by suicide cluster subject matter experts.

This process resulted in 245 articles. Duplicates were removed and abstracts were reviewed to exclude articles on suicide risk and protective factors that were not specific to clusters. All suicide-related social media articles were included even if they were not specifically about clusters because of the limited number of publications in this area. This process resulted in 166 articles that discussed cluster identification (included papers published during 1990–2022), risk and protective factors (2001–2022), prevention and response (2003–2022), and social media (2007–2022) (Figure). The quality of the literature was not formally assessed because of the relatively small number of publications found. Although findings from international settings would largely apply to the United States, some issues described might be unique to specific cultural environments.

**FIGURE:**
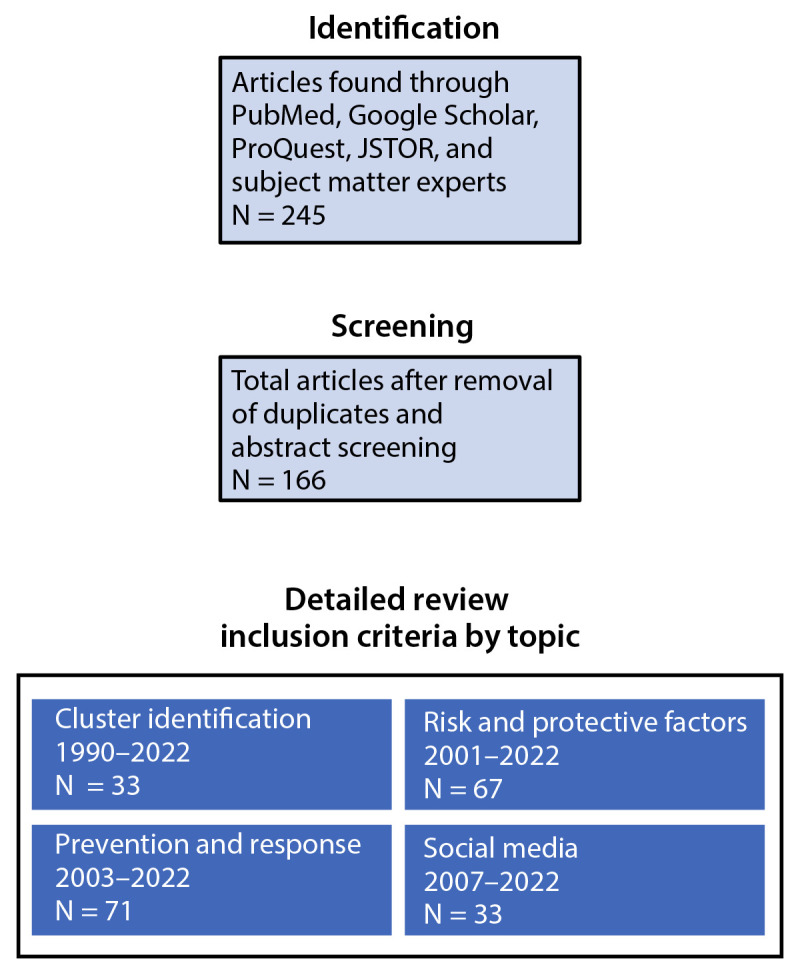
Number of articles identified, screened, and included during literature review of suicide clusters*, CDC guidance, 2024 * Article counts by topic area are not mutually exclusive. There were no geographic restrictions.

In addition, the team conducted an environmental scan that included eight internal Epidemiologic Assistance (Epi-Aid) reports from 2004–2018 documenting CDC support to local health jurisdictions to investigate and respond to a suspected suicide cluster. Other local investigations might have occurred without CDC involvement, and the team was not able to identify related reports to include in the review. The team also reviewed U.S. media reports during 2017–2022 to gather additional contextual information from communities that identified and responded to a suicide cluster but did not request Epi-Aid support from CDC. Media reports were identified through a Google News search of terms such as “suicide clusters united states.” A total of 14 news articles about clusters at the city, county, or university-level were identified and reviewed. Findings from reviews and scans might not be representative of all suicide clusters because of publication bias.

### Input from Subject Matter Experts

From December 2021 to May 2022, the team collected qualitative data through outreach to researchers and public health practitioners with suicide cluster subject matter expertise to gather input on lessons learned based on experiences responding to suspected suicide clusters; opportunities and challenges for using social media and the internet for suicide cluster identification, prevention, and response; and strengths and limitations of syndromic surveillance systems for identifying clusters. To do this, CDC participated in various virtual online meetings with grantees of three CDC-funded programs: Emergency Department Surveillance of Nonfatal Suicide-Related Outcomes (https://www.cdc.gov/suicide/programs/ed-snsro/index.html); Comprehensive Suicide Prevention (https://www.cdc.gov/suicide/programs/csp/index.html); and Injury Control Research Centers (https://www.cdc.gov/injury/erpo/icrc/index.html). In addition, CDC’s Center for Surveillance, Epidemiology, and Laboratory Services, which runs the National Syndromic Surveillance Program, provided information about how syndromic surveillance systems can be used for cluster detection and responses by communities. The team also attempted to connect with health departments who requested support from CDC for suicide cluster response during 2004–2018 to better understand key lessons from Epi-Aid investigations. Three health departments responded with feedback via email. In addition, the team conducted an online virtual topical focus group and individual virtual interviews with social media subject matter experts to discuss its role in suicide clustering. Social media subject matter experts were identified through suggestions from knowledgeable CDC team members and from author lists from published papers on this topic. Although all participants came from a convenience sample of subject matter experts known by the team, their input was critical to informing the guidance in this supplement. These discussions did not seek consensus from external subject matter experts on guidance or activities but were used to gather more information to inform CDC’s development of the reports in this supplement. Discussion questions used are presented (Box).

BOX Discussion questions used for subject matter expert* outreach, CDC guidance, 2024

**Table:** 

Emergency Department Surveillance of Nonfatal Suicide-Related Outcomes Grantees How are states using syndromic surveillance data for cluster detection? Do you review your syndromic data regularly to look for clusters and suicide attempts? How do you monitor changes in visit trends for suicide related visits (i.e., what data sources are used, what indicators or syndromes are tracked, and do you use temporal or spatial alerts)? How is a suicide cluster confirmed? Who has investigated a suicide cluster? What challenges did you experience when investigating suicide clusters? How are states responding to suicide clusters? What do you do after you confirm an increase in suicide related visits (e.g., follow up with the facility and send out public health messaging to the community)? How do you work with partners in your response (e.g., target prevention and control efforts, liaise with school counselors or school-based organizations, and who else do you work with)? What caveats are there when sharing counts/data for state and local partners? What’s the threshold for visit suppression? What can CDC include in updated suicide cluster guidance that would be helpful?
**Comprehensive Suicide Prevention Grantees** Has your health department investigated any recent suicide clusters? What challenges did you experience when investigating suicide clusters? What can CDC include in updated suicide cluster guidance that would be helpful?
**Injury Control Research Centers** Has your injury control research center performed research on suicide clusters, or assisted in the investigation of a suicide cluster? Based on your experiences, what do you think are the common challenges in researching or investigating suicide clusters? Are there any materials that CDC could develop or provide that would be helpful for future suicide cluster research or investigations?
**Health Departments That Requested CDC Assistance for Suicide Cluster Investigations** What alerted/indicated to you that there was a suicide cluster? What factors led to initiating a full investigation? How were suspected clusters confirmed? What happened once a cluster was identified and before you contacted CDC? What led to your decision to contact CDC? How were the findings and recommendations from the Epi-Aid used? What follow-up activities were implemented after the Epi-Aid investigation (e.g., changing environmental elements that might increase the likelihood of further suicides or suicide attempts and addressing potential long-term issues)? How well did it work? Looking back on the investigation, was there anything you wish was different? Was there anything missing from the investigation? Was there anything more that you needed? What worked well/didn’t work well? Is there anything else about that experience that you think might be useful to share with us?
**Social Media Experts** How can social media and other online web sources (e.g., Google Analytics) be used to identify clusters? How can social media be a part of the response to a cluster (risk factor/harmful effects, Papageno effect^†^)? What other considerations are there for social media and suicide clusters?

*Fewer than 10 respondents participated within each subject matter expert group.

^†^Responsible media reporting of suicide being a protective factor and making a positive contribution to prevention efforts by educating the public about coping strategies and treatment.

CDC used the information from the literature review, environmental scan, media review, and subject matter expert discussions to draft the reports and guidance in this supplement. Several additional external partners reviewed the drafts and provided high-level feedback, which CDC discussed and incorporated, as needed. *CDC Guidance for Community Assessment and Investigation of Suspected Suicide Clusters — United States, 2024* (*46*), describes guidance on responding to initial concerns for a suspected suicide cluster, confirming a cluster, and conducting an epidemiologic investigation. *CDC Guidance for Community Response to a Suicide Cluster *— *United States, 2024* (*48*), describes guidance on preparatory community action before cluster identification, direct response to the cluster, and action to help prevent the next cluster.

## Use of this Supplement

The guidance in this supplement is intended for public health practitioners, and state and local health departments. The guidance should not be considered explicit instructions to be followed by every community, but as suggestions on best practices. This information is meant to provide community leaders with a conceptual framework for assessing and investigating suspected clusters and developing their own suicide-cluster-response plans. These plans can be tailored to the particular needs, resources, and cultural characteristics of their communities.
